# Survival by first‐line therapy and prognostic group among men with metastatic castration‐resistant prostate cancer

**DOI:** 10.1002/cam4.7334

**Published:** 2024-06-22

**Authors:** Megan E. V. Caram, Kyle Kumbier, Phoebe A. Tsao, Jennifer Burns, Jordan B. Sparks, Kristian D. Stensland, Zachery R. Reichert, Joshi J. Alumkal, Brent K. Hollenbeck, Vahakn Shahinian, Alexander Tsodikov, Ted A. Skolarus

**Affiliations:** ^1^ Department of Internal Medicine University of Michigan Medical School Ann Arbor Michigan USA; ^2^ VA Health Services Research & Development, Center for Clinical Management and Research VA Ann Arbor Healthcare System Ann Arbor Michigan USA; ^3^ Department of Urology University of Michigan Ann Arbor Michigan USA; ^4^ Department of Urology Massachusetts General Hospital Boston Massachusetts USA; ^5^ Department of Biostatistics, School of Public Health University of Michigan Ann Arbor Michigan USA; ^6^ Department of Surgery, Urology Section University of Chicago Chicago Illinois USA

**Keywords:** novel therapies, prognosis, prostate cancer

## Abstract

**Introduction:**

Metastatic castration‐resistant prostate cancer (mCRPC) is a heterogeneous disease with prognoses varying from months to years at time of castration‐resistant diagnosis. Optimal first‐line therapy for those with different prognoses is unknown.

**Methods:**

We conducted a retrospective cohort study of men in a national healthcare delivery system receiving first‐line therapy for mCRPC (abiraterone, enzalutamide, docetaxel, or ketoconazole) from 2010 to 2017, with follow‐up through 2019. Using commonly drawn prognostic labs at start of mCRPC therapy (hemoglobin, albumin, and alkaline phosphatase), we categorized men into favorable, intermediate, or poor prognostic groups depending on whether they had none, one to two, or all three laboratory values worse than designated laboratory cutoffs. We used Kaplan–Meier methods to examine prostate specific antigen (PSA) progression‐free and overall survival (OS) according to prognostic group and first‐line therapy, and multivariable cox regression to determine variables associated with survival outcomes.

**Results:**

Among 4135 patients, median PSA progression‐free survival (PFS) was 6.9 months (95% confidence interval [CI] 6.6–7.3), and median OS 18.8 months (95% CI 18.0–19.6), ranging from 5.7 months (95% CI 4.8–7.0) in the poor prognosis group to 31.3 months (95% CI 29.7–32.9) in the favorable group. OS was similar regardless of initial treatment received for favorable and intermediate groups, but worse for those in the poor prognostic group who received ketoconazole (adjusted hazard ratio 2.07, 95% CI 1.2–3.6). PSA PFS was worse for those who received ketoconazole compared to abiraterone across all prognostic groups (favorable HR 1.76, 95% CI 1.34–2.31; intermediate HR 1.78, 95% CI 1.41–2.25; poor HR 8.01, 95% CI 2.93–21.9).

**Conclusion:**

Commonly drawn labs at mCRPC treatment start may aid in predicting survival and response to therapies, potentially informing discussions with care teams. First‐line treatment selection impacts disease progression for all men with mCRPC regardless of prognostic group, but impacted OS only for men with poor prognosis at treatment start.

## Graphical abstract

Hemoglobin, albumin, and alkaline phosphatase, used to categorize patients with metastatic castration resistant prostate cancer at start of new treatment into prognostic groups, was strongly predictive of survival, but survival did not vary substantially based on which treatment patients received within prognostic groups.

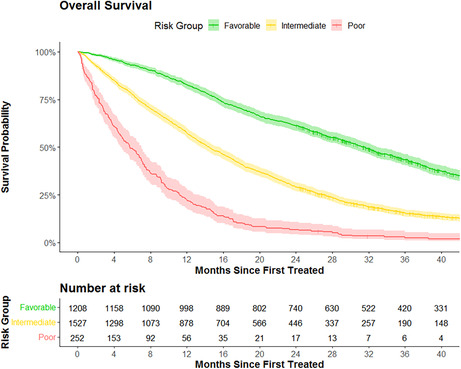

## 1 INTRODUCTION

The treatment landscape for metastatic castration‐resistant prostate cancer (mCRPC) has dramatically changed over the past two decades. In 2004, docetaxel became standard of care after demonstrating improved survival compared to steroids and mitoxantrone, treatments that provided symptomatic improvement.^1^ Ketoconazole was also commonly used to palliate symptoms and lower castration‐resistant prostate specific antigen (PSA) levels, despite an unknown benefit to survival in randomized trials.^2^, ^3^, ^4^, ^5^ Prior to 2013, ketoconazole was used more commonly than docetaxel to treat men with mCRPC.^6^ When abiraterone and enzalutamide were approved for first‐line mCRPC treatment a decade after docetaxel, these novel therapies were not compared to the standard of care docetaxel, or the commonly used off‐label ketoconazole, but were compared to control arms of prednisone and placebo, respectively.^7^, ^8^ Therefore, whether the novel mCRPC therapies improved overall survival (OS) in real‐world settings compared to the prior contemporary standard of care (ketoconazole or docetaxel) remains unknown.

Furthermore, while mCRPC is a fatal condition, prognosis is variable with some patients surviving <6 months and others several years, calling into question whether benefits from first‐line therapies are experienced the same among men with different prognoses. For example, should patients with more aggressive poor prognostic disease receive docetaxel first‐line rather than abiraterone or enzalutamide.^9^, ^10^, ^11^ Laboratory values, including hemoglobin, albumin, and alkaline phosphatase (ALP) have been included in prior prognostic models for patients with mCRPC among other factors such as lactate dehydrogenase (LDH), performance status, visceral disease, or circulating tumor fraction.^9^, ^10^, ^12^ However, the utilization of validated prognostic models is low due to their complexity and the fact that all variables are not available at treatment initiation (e.g., LDH is rarely checked). A model utilizing commonly drawn baseline values (i.e., hemoglobin, albumin, ALP) that are routinely checked in patients at start of therapy would be welcome and may allow better matching of individual disease risk with treatment.

In this context, we conducted a study of men with prostate cancer that progressed from castration‐sensitive to mCRPC to better understand outcomes of typical first‐line mCRPC treatment options, while creating a usable prognostic model within a national healthcare delivery system. We used common laboratory values to categorize patients into favorable, intermediate, and poor risk prognostic groups, and examined whether the most used first‐line therapies for mCRPC (abiraterone, enzalutamide, docetaxel, or ketoconazole) were associated with differences in OS or PSA progression‐free survival (PFS) within and across prognostic groups. Our findings may help inform discussions about prognosis and treatment selection for providers and their patients with mCRPC.

## 2 MATERIALS AND METHODS

### 2.1 Cohort identification

To conduct this study, we used data from the Veterans Health Affairs (VA) national healthcare system (130 local healthcare systems within 18 regional networks) VA Corporate Data Warehouse (CDW). VA CDW includes claims, pharmacy, laboratory, and medical record data from all VA facilities. Patients in our final cohort had a diagnosis code for prostate cancer using International Classification of Diseases, Ninth Revision (ICD‐9) code 185 for 2010 through 2015, and ICD‐10 code C61 for 2016 and 2017, had mCRPC, and were prescribed abiraterone, enzalutamide, docetaxel, or ketoconazole between 2010 and 2017 as their first‐line therapy after they were determined to have castration‐resistance. We initially also included mitoxantrone, sipuleucel‐T, radium‐223, and cabazitaxel as treatments of interest, but since <1% of patients received these therapies first‐line, we excluded them from final analyses. Follow‐up was through 2019. We confirmed metastatic disease using a validated natural language processing algorithm within the national healthcare system electronic record with over 91% sensitivity and 97% specificity.^13^ Castration‐resistance was confirmed through previously published methods that required rising PSA levels while receiving androgen deprivation therapy for at least 6 months within the VA.^6^ Patients who received one of the four therapies of interest (abiraterone, enzalutamide, docetaxel, ketoconazole) before study start were excluded since they were not considered to be receiving first‐line mCRPC treatment. We chose 2017 as the last year of measured treatment start for this mCRPC study because abiraterone and enzalutamide became standard of care in an earlier castration‐sensitive setting in 2018 and 2019.^14^, ^15^, ^16^, ^17^ Restricting our study period to before this shift minimizes misclassification and selection biases associated with mCRPC designation and outcomes. The CONSORT diagram for inclusion is included as a Supplemental Figure—Data S1.

### 2.2 mCRPC prognostic groups

To categorize patients into mCRPC prognostic groups, we restricted the cohort to those with hemoglobin, albumin, and ALP laboratory tests checked in the 60 days prior to start of mCRPC therapy. We chose hemoglobin, albumin, and ALP based on previous data showing their prognostic value and because they are routinely checked in patients prior to therapy start.^18^ We did not include prognostic variables in the literature that are rarely checked in practice (e.g., LDH checked in <20% of patients), and those that were not available in our dataset such as visceral metastases and performance status.^9^, ^10^ PSA and PSA doubling time were included as independent variables, but not used to define prognostic group categories since PSA was used on a logarithmic scale in prior prognostic studies and had less impact on prognosis than hemoglobin, albumin, and ALP.^18^ We chose to categorize hemoglobin, albumin, and ALP laboratory values rather than using continuous values to simplify implementation into practice. Multiple factors were considered in choosing our cutoffs for the three prognostic groups, favorable, intermediate, and poor risk. First, prior prognostic studies included patients treated in clinical trials, but most patients in real‐world practice tend to be less fit and healthy than those included in clinical trials; for example, anemia is prevalent in men with advanced prostate cancer on androgen deprivation therapy, yet prior prognostic studies include clinical trial patients who have a relatively normal median hemoglobin of 12.8 g/dL.^19^ The median hemoglobin and interquartile range for patients in our cohort was 12.1 (IQR 10.6–13.3). In addition, there is some variability of laboratory normal limits among facilities (e.g., laboratory facility upper limit of normal for ALP ranged from 120 to 180 U/L). Finally, we considered cutoffs that are clinically important to clinicians when observed in patients with multiple comorbid conditions. Taking these conditions into account, and the interquartile ranges of these laboratory values in our cohort (Table S1), we defined the favorable risk group as patients with hemoglobin >10 g/dL, ALP <200 International U/L, and albumin >3.5 g/dL at the start of mCRPC therapy. The intermediate risk group included patients with one or two of these laboratory values outside the favorable range, and the poor risk group included those in whom all three values were outside the favorable range.

### 2.3 Outcomes

Our primary outcome was OS in months from start of first‐line mCRPC therapy by pharmacy fill date or docetaxel infusion. Our secondary outcome was PSA PFS while on first‐line therapy, defined as 25% increase in PSA from baseline if PSA never declined on therapy, or from PSA nadir if PSA declined on therapy.^20^ For PSA PFS, we excluded patients without at least two PSA values during first‐line treatment, resulting in a smaller sample size. We also characterized subsequent treatments patients received after first‐line therapy.

### 2.4 Independent variables

We adjusted for patient sociodemographic, disease‐specific, and facility variables in our multivariable models (facility variables described in detail in Table S2 legend). We also included year of treatment start since improvement in later lines of mCRPC therapy could affect overall prostate cancer outcomes.

### 2.5 Analysis

We used descriptive and univariate statistics to characterize our cohort according to prognostic groups. We conducted Kaplan–Meier analyses to determine OS and PSA PFS for patients in different prognostic groups at the start of therapy and for the different first‐line therapies stratified by prognostic group. Since the prognostic group was a strong predictor of survival outcomes, we stratified our multivariable Cox regression analyses, fitting models for each of the three prognostic groups.

This study was approved by the Veterans Affairs and University of Michigan Institutional Review Boards. Informed consent was waived since this study used secondary coded private data and only restricted variables. This study followed the Strengthening the Reporting of Observational Studies in Epidemiology guidelines.^21^

## 3 RESULTS

We identified 4135 patients who started ketoconazole, docetaxel, abiraterone, or enzalutamide as first‐line treatment for mCRPC between 2010 and 2017. Table 1 shows sociodemographic and disease‐specific variables. Among those with laboratory data available to categorize into prognostic cohorts (*n* = 2987, 72% total cohort), 40% met criteria for the favorable risk group, 51% intermediate, and 8% for poor risk. Those without complete laboratory data and thus not included in the prognostic groups did not differ appreciably by disease status, age, race, or facility (Table S2). Median age was 73, 55% had a Charlson Comorbidity Index (CCI) 0, and almost one third of our cohort identified as Black.

**TABLE 1:** Patient characteristics of full cohort and prognostic groups.

Variables	Full cohort (*n* = 4135)	Prognostic groups (*n* = 2987)			
		Favorable (*n* = 1208, 40%)	Intermediate (*n* = 1527, 51%)	Poor (*n* = 252, 8%)	Group Comparison *p*‐value
Drug (*N*, % by strata)					
Abiraterone	1731 (42%)	540 (45%)	638 (42%)	94 (37%)	<0.001
Enzalutamide	784 (19%)	232 (19%)	275 (18%)	30 (12%)	
Docetaxel	762 (18%)	218 (18%)	344 (23%)	89 (35%)	
Ketoconazole	858 (21%)	218 (18%)	270 (18%)	39 (15%)	
Age at first drug, years (median, IQR)	73 (66, 82)	71 (66, 79)	75 (67, 83)	71 (65, 81)	<0.001
Race (*N*, % by strata)					
White	2631 (64%)	892 (74%)	869 (57%)	126 (50%)	<0.001
Black	1181 (29%)	234 (19%)	545 (36%)	107 (42%)	
Other	68 (2%)	17 (1%)	30 (2%)	3 (1%)	
Unknown	255 (6%)	65 (5%)	83 (5%)	16 (6%)	
Charlson Comorbidity Index (*N*, % by strata)					
0	2266 (55%)	750 (62%)	732 (48%)	109 (43%)	<0.001
1	913 (22%)	253 (21%)	374 (24%)	56 (22%)	
2+	956 (23%)	205 (17%)	421 (28%)	87 (35%)	
Starting PSA, ng/mL (median, IQR)	46 (15, 144)	25 (10, 68)	63 (21, 188)	344 (116, 810)	<0.001
PSA doubling time, months (median, IQR)	6.0 (4.7, 8.1)	6.3 (5.1, 8.5)	5.9 (4.6, 8.0)	5.1 (4.0, 6.4)	<0.001
PSA doubling time (*N*, % by strata)					
<3 months	235 (6%)	48 (4%)	101 (7%)	21 (8%)	<0.001
3 to <6 months	1804 (44%)	480 (40%)	685 (45%)	157 (62%)	
6 to <10 months	1476 (36%)	466 (39%)	510 (33%)	54 (21%)	
≥10 months	620 (15%)	214 (18%)	231 (15%)	20 (8%)	
Year at time of first treatment (*N*, % by year)					
2010	346	108 (31%)	118 (34%)	27 (8%)	<0.001
2011	510	123 (24%)	182 (36%)	37 (7%)	
2012	457	99 (22%)	190 (42%)	42 (9%)	
2013	468	122 (26%)	188 (40%)	30 (6%)	
2014	548	172 (31%)	202 (37%)	33 (6%)	
2015	558	160 (29%)	204 (37%)	28 (5%)	
2016	605	209 (35%)	208 (34%)	31 (5%)	
2017	643	215 (33%)	235 (37%)	24 (4%)	

Abbreviations: IQR, interquartile range; PSA, prostate specific antigen.

*Note*: Patient characteristic comparison among different prognostic groups. Prognostic groups were defined as: Favorable group included patients who had hemoglobin >10 g/dL, albumin >3.5 g/dL, and alkaline phosphatase <200 International U/L at the start of metastatic castration‐resistant prostate cancer therapy; Intermediate group included patients with one or two of these laboratory values that was out of the favorable range; poor prognostic group included patients for whom all three values were out of the favorable range.

We found differences in clinical and non‐clinical variables among patients in the different prognostic groups (Table 1). Patients in the poor prognostic group had a CCI 2+ (35%) versus 28% in intermediate risk and 17% in favorable risk, *p* < 0.001. We also observed a strong relationship between prognostic groups and disease variables such as PSA and PSA doubling time. Patients in the poor prognostic group had substantially higher PSA at the start of mCRPC therapy (median PSA 344 ng/mL poor risk, 63 ng/mL intermediate risk, 25 ng/mL favorable risk, *p* < 0.001). Similarly, median PSA doubling time was shortest in the poor prognostic group and longest in the favorable group, suggesting the laboratory thresholds we used to define the poor prognostic group did in fact reflect disease aggressiveness (Table 1).

### 3.1 Kaplan–Meier analyses

**FIGURE 1:**
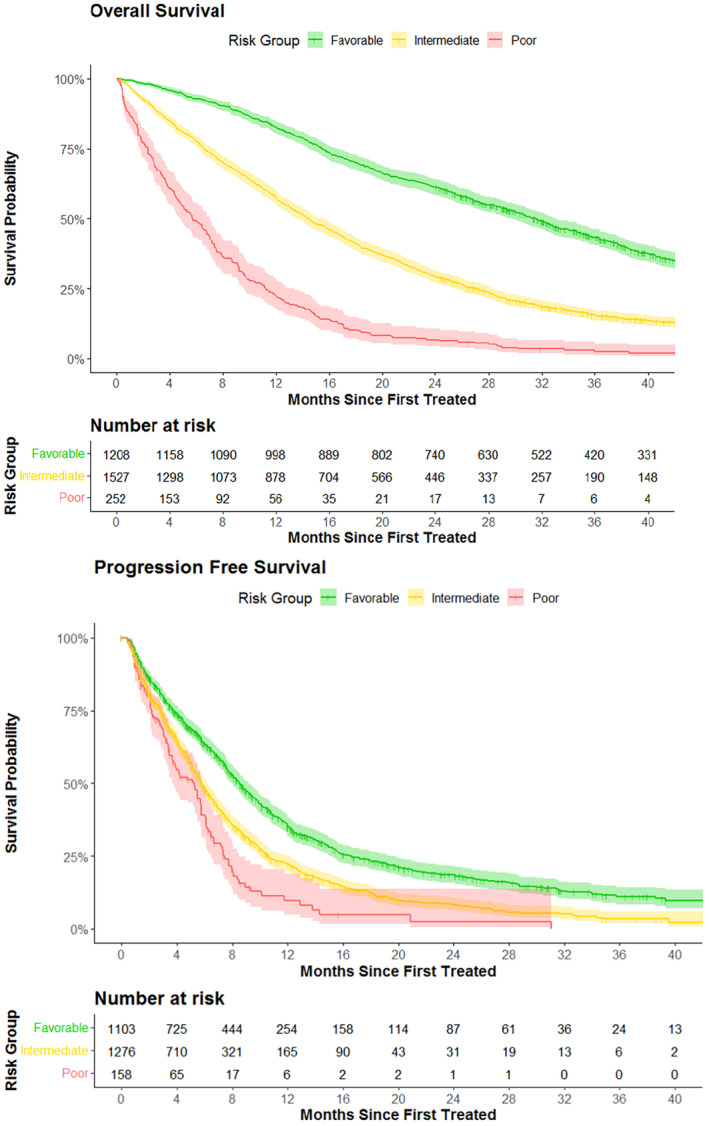
Overall survival and PSA progression‐free survival by prognostic group (*n* = 2987). PSA, prostate specific antigen. Overall survival was from time of metastatic castration‐resistant prostate cancer therapy to death or end of study duration. PSA progression‐free survival was defined as the time from start of first treatment to the first PSA lab that represented at least a 25% increase in PSA from nadir or baseline. Prognostic groups were defined as: favorable group included patients who had hemoglobin >10 g/dL, albumin >3.5 g/dL, and alkaline phosphatase <200 International U/L at the start of metastatic castration‐resistant prostate cancer therapy; Intermediate group included patients with one or two of these laboratory values that was out of the favorable range; Poor prognostic group included patients for whom all three values were out of the favorable range.

In the entire cohort, median OS was 18.8 months (95% CI 18.0–19.6 months), and median PSA PFS was 6.9 months (95% confidence interval [CI] 6.6–7.3 months). Median OS varied substantially among prognostic groups from 31.3 months (95% CI 29.7–32.9) in the favorable group to 5.7 months (95% CI 4.8–7.0) in the poor risk group (*p* < 0.001) (Figure 1). Median PSA PFS ranged from 8.5 months, 95% CI 8.0–9.2 in the favorable group to 5.3 months, 95% CI 3.7–5.8 in the poor risk group (*p* < 0.001) (Figure 1).

### 3.2 OS and PSA PFS by first‐line therapy

**FIGURE 2:**
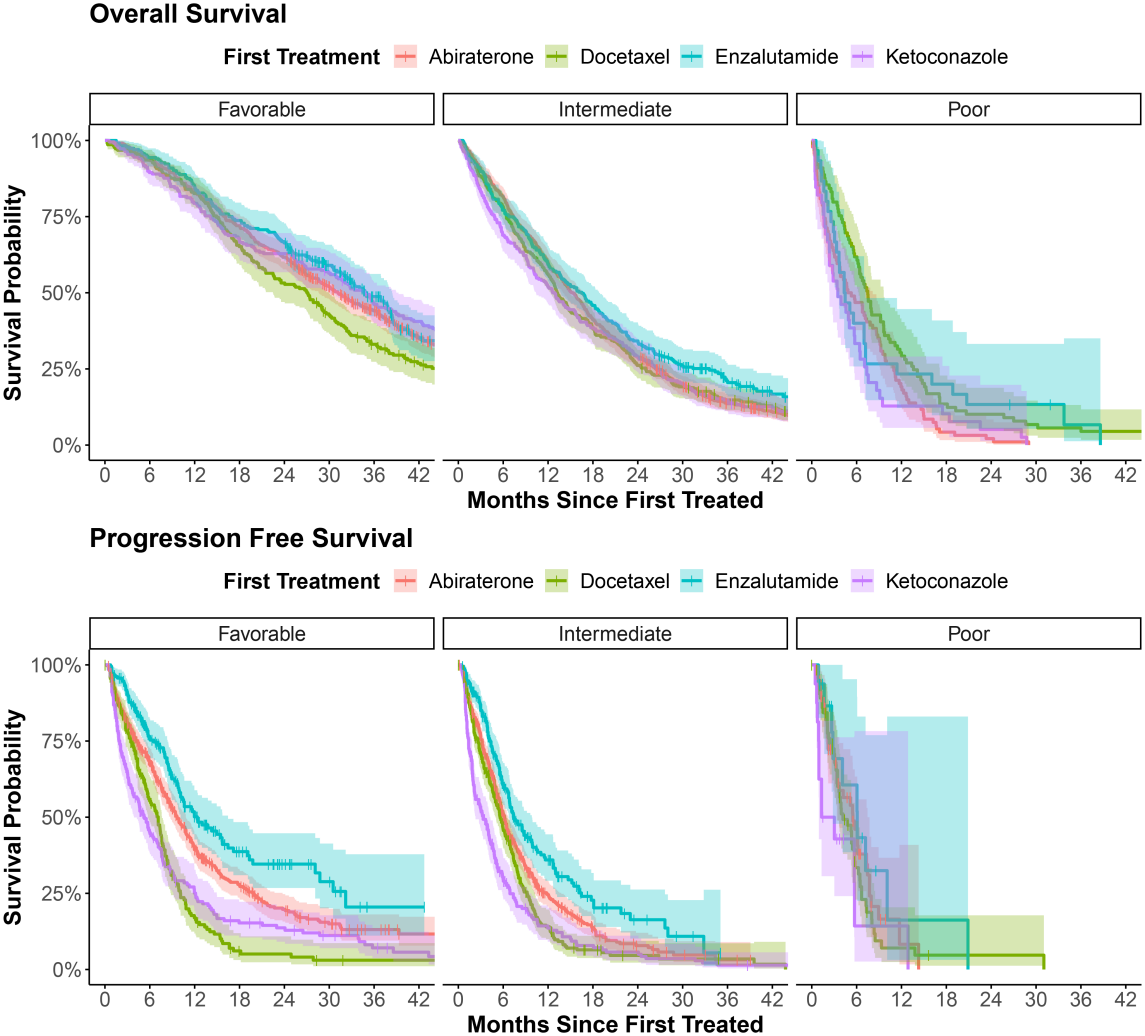
Overall survival and PSA progression‐free survival within prognostic groups by first‐line therapy received (*n* = 2987). PSA, prostate specific antigen. Overall survival was from time of metastatic castration‐resistant prostate cancer therapy to death or end of study duration. PSA progression‐free survival was defined as the time from start of first treatment to the first PSA lab that represents at least a 25% increase in PSA from nadir or baseline. Prognostic groups were defined as: Favorable group included patients who had hemoglobin >10 g/dL, albumin >3.5 g/dL, and alkaline phosphatase <200 International U/L at the start of metastatic castration‐resistant prostate cancer therapy; Intermediate group included patients with one or two of these laboratory values that was out of the favorable range; Poor prognostic group included patients for whom all three values were out of the favorable range.

Since our prognostic groups had such a strong impact on outcomes and the exposure (first‐line treatment), subsequent analyses were stratified by prognostic group. In unadjusted Kaplan Meier analyses, median OS by first‐line therapy varied within and among prognostic groups (Figure 2). In the favorable risk group, median OS ranged from 27.1 months (95% CI 22.5–30.0) for patients who received docetaxel to 35.0 months (95% CI 31.8–38.4) for enzalutamide (*p* = 0.001). In the intermediate risk group, median OS ranged from 12.8 months (95% CI 10.4–15.7) for patients who received ketoconazole to 16.5 months (95% CI 13.7–18.8) for enzalutamide (*p* = 0.021). In the poor risk group, median OS ranged from 3.5 months (95% CI 2.4–6.4) for patients who received ketoconazole to 7.5 months (95% CI 6.3–9.6) for docetaxel (*p* = 0.006) (Figure 2).

In each of the prognostic groups, median PSA PFS was shortest for patients who received first‐line ketoconazole and longest for patients who received enzalutamide. In the favorable risk group, median PSA PFS ranged from 5.3 months (95% CI 3.9–7.0) for ketoconazole to 12.4 months (95% CI 10.1–16.5) for enzalutamide (*p* < 0.001). In the intermediate risk group, median PSA PFS ranged from 3.3 months (95% CI 2.3–4.3) for ketoconazole to 7.4 months (95% CI 6.7–9.8) for enzalutamide (*p* < 0.001). Due to inadequate sample size, we were unable to compare PSA PFS for first‐line treatment in the poor risk group. Because of residual confounders that could affect outcomes even after stratification, such as year of treatment, facility, comorbid conditions, and age, multivariable adjustment was performed.

### 3.3 Multivariable cox regression analyses by prognostic group

Using multivariable cox regression analyses, increasing age and comorbidity were associated with worse OS in all prognostic groups, but were not associated with PSA PFS (Table 2; Table S3). More aggressive disease variables (higher PSA and shorter PSA doubling time) were associated with shorter OS in the favorable and intermediate risk prognostic groups but not in the poor prognostic group (Table 2), which may have been explained by the smaller poor risk sample size. Importantly, year and facility type did not have an appreciable effect on OS or PSA PFS.

**TABLE 2:** Multivariable cox regression analysis for overall survival.

Independent variables	Hazard ratios (95% confidence intervals)		
	Stratified models (# of observations in full adjusted model)		
	Favorable (*n* = 1106)	Intermediate (*n* = 1414)	Poor (*n* = 233)
First‐line treatment			
Abiraterone	Ref.	Ref.	Ref.
Enzalutamide	0.94 (0.75, 1.17)	0.88 (0.74, 1.04)	0.68 (0.41, 1.14)
Docetaxel	1.13 (0.90, 1.41)	1.05 (0.88, 1.25)	0.89 (0.59, 1.34)
Ketoconazole	0.97 (0.75, 1.24)	1.07 (0.88, 1.30)	2.07 (1.21, 3.57)
Age (years)	1.01 (1.01, 1.02)	1.02 (1.01, 1.02)	1.02 (1.00, 1.04)
Race			
White	Ref.	Ref.	Ref.
Black	0.69 (0.57, 0.84)	0.75 (0.65, 0.86)	1.05 (0.72, 1.54)
Other	0.63 (0.34, 1.16)	0.67 (0.45, 0.99)	1.30 (0.37, 4.55)
Charlson Comorbidity Index			
0	Ref.	Ref.	Ref.
1	1.43 (1.20, 1.71)	1.10 (0.96, 1.27)	1.52 (0.99, 2.34)
2+	1.58 (1.31, 1.90)	1.28 (1.12, 1.46)	1.53 (1.05, 2.22)
Starting PSA (log scale)^a^	1.22 (1.15, 1.28)	1.13 (1.08, 1.17)	1.12 (0.99, 1.27)
PSA doubling time			
<3 months	Ref.	Ref.	Ref.
3–6 months	0.79 (0.55, 1.12)	1.04 (0.82, 1.31)	1.10 (0.61, 1.97)
6–10 months	0.59 (0.41, 0.83)	0.71 (0.56, 0.91)	0.84 (0.45, 1.57)
>10 months	0.53 (0.36, 0.78)	0.59 (0.45, 0.78)	0.55 (0.27, 1.13)
Start year			
2010	Ref.	Ref.	Ref.
2011	0.96 (0.72, 1.28)	0.78 (0.61, 1.00)	1.24 (0.66, 2.31)
2012	1.11 (0.81, 1.52)	0.89 (0.69, 1.14)	1.66 (0.91, 3.03)
2013	0.78 (0.56, 1.07)	0.87 (0.66, 1.14)	2.12 (1.11, 4.03)
2014	0.97 (0.71, 1.33)	0.84 (0.64, 1.10)	1.99 (1.00, 3.96)
2015	0.96 (0.68, 1.35)	0.93 (0.70, 1.23)	1.34 (0.69, 2.60)
2016	0.92 (0.66, 1.28)	0.81 (0.61, 1.07)	1.91 (0.94, 3.89)
2017	0.78 (0.54, 1.12)	0.85 (0.64, 1.13)	1.22 (0.58, 2.53)
Distance to facility (per 10 miles)	1.00 (1.00, 1.01)	1.00 (0.99, 1.00)	1.00 (0.99, 1.01)
Urban or Rural			
Urban	Ref.	Ref.	Ref.
Rural	0.93 (0.80, 1.09)	1.01 (0.89, 1.15)	0.98 (0.65, 1.46)

Abbreviation: PSA, prostate specific antigen.

*Note*: The distance from the patient's home zip code to the treating facility and urban/rural status of the facility are variables shown here. Urban/rural status was determined by the patient's zip. Hazard ratios were also adjusted for facility level covariates including facility complexity, proportion of Black patients, proportion of patients with diagnosed prostate cancer who were treated for advanced disease, staffing of hematology/oncology and urology physicians (based on full‐time equivalents), and whether the facility was an early adopter of novel oral therapies abiraterone and enzalutamide. These other variables not shown were not significant.

^a^ PSA was log‐transformed for this multivariable analysis to make it more normally distributed. Prognostic groups were defined as: favorable group included patients who had hemoglobin >10 g/dL, albumin >3.5 g/dL, and alkaline phosphatase <200 International U/L at the start of metastatic castration‐resistant prostate cancer therapy; Intermediate group included patients with one or two of these laboratory values that was out of the favorable range; Poor prognostic group included patients for whom all three values were out of the favorable range.

After adjustment, there was no significant difference in OS among patients receiving any of the first‐line therapies in the favorable and intermediate risk groups (Table 2). However, men in the poor prognostic group had worse OS if they received ketoconazole first‐line rather than abiraterone (HR 2.07, 95% CI 1.21–3.57) (Table 2). First‐line docetaxel use was not associated with improved OS in the poor prognostic group.

Compared to receiving abiraterone first‐line, receiving docetaxel in the favorable prognostic group was associated with worse PSA PFS (HR 1.67, 95% CI 1.30–2.15). Enzalutamide in the favorable and intermediate prognostic groups was associated with a better PSA PFS (HR 0.70, 95% CI 0.55–0.90 favorable; HR 0.78, 95% CI 0.62–0.97 intermediate) (Table S3). Ketoconazole was associated with worse PSA PFS across all prognostic groups (favorable HR 1.76, 95% CI 1.34–2.31; intermediate HR 1.78, 95% CI 1.41–2.25; poor HR 8.01, 95% CI 2.93–21.9) (Table S3).

### 3.4 Subsequent therapy

**FIGURE 3:**
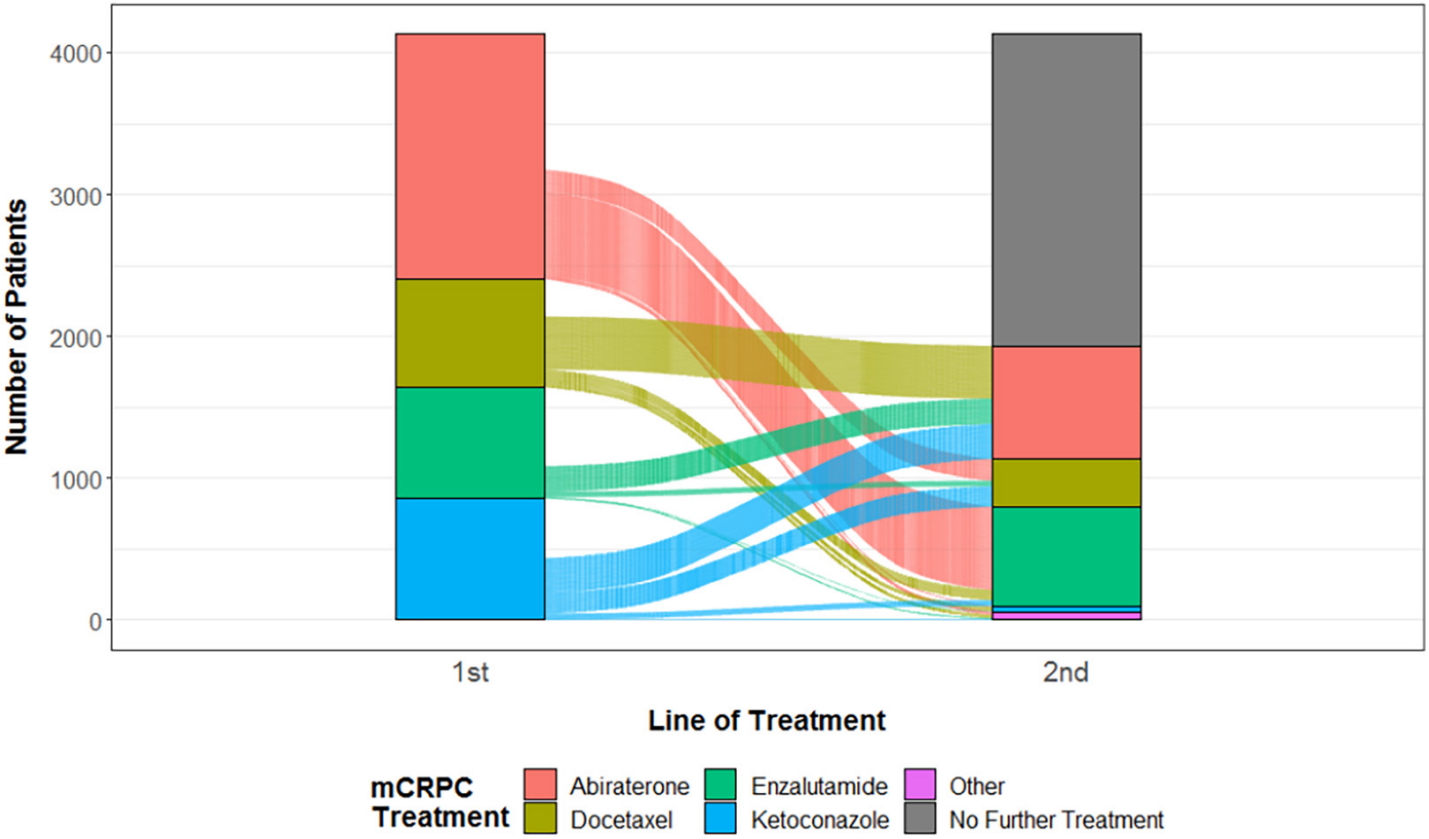
Subsequent second‐line therapy. The first bar represents the frequency of first‐line therapies used and the second bar represents the second line therapy used. The lines connecting the two bars indicate individual patients who went on to receive each second line therapy.

As shown in Figure 3 and described further in Table S4, approximately half of patients (47%) received second‐line mCRPC therapy, ranging from 65% of patients who received first‐line docetaxel to 29% of patients receiving first‐line enzalutamide. Most second‐line therapy was either abiraterone or enzalutamide.

## 4 DISCUSSION

Using three routinely checked laboratory variables (hemoglobin, albumin, ALP) at the start of mCRPC therapy to categorize patients into prognostic groups, we found a two‐year difference in OS between patients grouped in the favorable versus poor prognostic groups, suggesting this simple, threshold‐based prognostic tool could help clinicians more clearly understand the heterogeneity of mCRPC and discuss prognosis with their patients. After stratifying patients into three different prognostic groups and adjusting for patient factors, facility, and year of treatment, we found first‐line therapy selection did not impact OS in those with favorable or intermediate prognosis. However, in those with poor prognosis at start of therapy, receiving ketoconazole was associated with worse OS. First‐line therapy selection did impact PSA PFS across all prognostic groups.

Rigorous studies have used training, testing, and validation cohorts to develop prognostic algorithms for patients with mCRPC, including variables such as site of metastatic disease, performance status, and laboratory values (e.g., hemoglobin, ALP, LDH).^10^, ^18^, ^19^, ^22^ However, it is difficult to generalize these algorithms to the general prostate cancer population as they were tested and validated in patients enrolled in clinical trials, patients who differ from the general population. For example, a recent study testing a prognostic algorithm included 8083 patients from Phase III trials. Although rigorous and comprehensive, only 5% of patients were Black, median hemoglobin was 12.8 g/dL, and median ALP 119 U/L. In contrast, 30% of our cohort was Black, and the majority (>60%) of patients in our cohort had hemoglobin or ALP that would have disqualified them from being included in the prognostic algorithm testing of that study. Furthermore, laboratory values in prior studies examining prognosis are usually measured on a continuous scale with wide ranges, and typically require an online calculator. Some algorithms also require variables not typically drawn in routine care, such as LDH. When designing our prognostic groups, we attempted to use the most checked, impactful laboratory tests identified in prior studies that would be readily available to clinicians and simplified them categorically for clinical practice with easy to remember thresholds that don't require an online calculator or tool.

Choosing therapy for men with mCRPC in the past decade has been largely based on physician and patient preference as there have been no randomized controlled trials comparing efficacy of different therapies in the first‐line setting. One large observational study done through a prostate cancer registry compared the efficacy of first‐line abiraterone, enzalutamide, and docetaxel in patients with mCRPC and found no significant difference in OS, similar to our study findings (albeit in a smaller cohort of 1583 patients).^23^ However, although the registry study stratified patients by visceral disease and other comorbid conditions, it did not use prognostic groups to account for the heterogeneity of mCRPC. In addition, the registry study did not include ketoconazole, which has remained on guidelines for those without access to abiraterone or enzalutamide. Ketoconazole, like abiraterone, inhibits CYP17A1, an enzyme required in the synthesis of androgens. It is through inhibition of CYP17A1 that abiraterone exerts its prostate cancer treatment effect, yet ketoconazole is thought to be less selective, less potent, and has never been shown to demonstrate the same survival benefits as abiraterone.^24^ Smaller studies have found ketoconazole to reduce PSA in 50% of patients in the first‐line mCRPC setting, but have not demonstrated a survival benefit.^4^ No registrational Phase 3 study in the first line (docetaxel, abiraterone, and enzalutamide) utilized ketoconazole in the control arm, so a direct comparison of ketoconazole to abiraterone or enzalutamide has not been done and no first‐line mCRPC retrospective studies have included it. A retrospective study done in the second‐line docetaxel‐refractory mCRPC setting in an international database compared 26 patients receiving ketoconazole to 140 patients receiving abiraterone and showed progression free survival and OS were both worse in patients receiving ketoconazole.^25^ Acknowledging the fact that not all patients have access to abiraterone and enzalutamide, mostly for financial reasons, guidelines have previously recommended ketoconazole still be considered for patients who could not access abiraterone or enzalutamide.^26^, ^27^ Our data includes over 800 patients who received ketoconazole in the first‐line mCRPC setting and confirmed that ketoconazole is inferior to abiraterone in delaying PSA progression for most patients with prostate cancer. Thus, work to decrease barriers to abiraterone and enzalutamide remains paramount.

The heterogeneity of mCRPC prognosis is apparent even in clinical trials where the OS of patients included in first‐line mCRPC clinical trials varies considerably. Median OS for men with mCRPC receiving first‐line docetaxel in the TAX 327 was 19 months, whereas median OS was almost twice as long at 35 months in PREVAIL that investigated first‐line enzalutamide in patients with mCRPC.^1^, ^10^, ^23^, ^28^ In addition, PREVAIL required patients to be asymptomatic or minimally symptomatic, which typically selects patients with the best prognosis. As stated previously, our cohort differed significantly from patients enrolled in clinical trials; 60% of our patients had laboratory values that placed them in the intermediate or poor prognostic group, which would have excluded them from participation in clinical trials. Nevertheless, the median OS in our total cohort of patients was 18.8 months, comparable to the OS of patients on the TAX 327 study. However, interestingly, the median OS of our favorable risk group was 31.3 months, comparable to the survival of patients in the PREVAIL trial where median survival was 35 months for those who received enzalutamide.^8^

There are important strengths and limitations in this study. First, this was the largest study to analyze comparative effectiveness of therapies in a national real‐world cohort of men with mCRPC in the United States. Although a common critique of studies conducted in the Veterans Health Administration (VA) is the concern about generalizability of the results to populations outside the VA, it is important to note that the VA population encompasses men who are inadvertently but nonetheless systematically excluded from clinical trials, namely Black men and men of lower socioeconomic status who are disproportionately affected by prostate cancer. Most clinical trials and studies examining prognostic variables in patients include <5% Black patients.^19^ Understanding outcomes for men who are underrepresented in clinical trials is important if the oncology community is to understand patient outcomes in the real‐world setting. In addition, reduced financial toxicity experienced by men receiving care in the VA allows a comparison of clinical effectiveness of therapies, excluding out‐of‐pocket cost as a determining factor. The limited variables we used to construct our prognostic groups is also an important limitation. Our laboratory estimates were conservative and chosen to reflect feasible cutoffs generalizable to all VA facilities. However, the conservative thresholds we used resulted in a small poor risk group which may have impacted our ability to detect further differences. In addition, we may not have captured subsequent therapies if patients left the VA for treatments; however, because the frequency of second‐line therapy in our cohort was similar to prior studies,^23^ and the VA provides low‐cost prescriptions, we think the number of patients leaving the VA for subsequent therapy is likely to be minimal. Finally, our prognostic groups were created with the intention of stratifying patients to compare effectiveness of first‐line treatments in a heterogenous cohort; thus, these simplified prognostic groups have not been validated using training and validation data sets, which we hope to study in future studies.

## 5 CONCLUSION

Routinely drawn laboratory tests may be useful in informing clinicians about prognosis in a heterogeneous national sample of patients with mCRPC. It is notable that our retrospective analysis showed first‐line treatment selection of abiraterone, enzalutamide, docetaxel, or ketoconazole did not appear to impact OS for the majority of patients (i.e., favorable and intermediate risk). Nevertheless, it is clear that other measures such as PSA PFS were inferior in patients receiving ketoconazole. Due to the retrospective nature of our study, abiraterone, enzalutamide, or docetaxel, which have been proven to improve OS in randomized controlled trials, still remain standard of care.

## AUTHOR CONTRIBUTIONS

**Megan E. V. Caram:** Conceptualization (lead); funding acquisition (lead); methodology (equal); supervision (lead); visualization (equal); writing – original draft (lead); writing – review and editing (lead). **Kyle Kumbier:** Data curation (equal); formal analysis (equal); investigation (equal); methodology (lead); software (equal); validation (lead); writing – review and editing (equal). **Phoebe A. Tsao:** Conceptualization (equal); visualization (equal); writing – review and editing (equal). **Jennifer Burns:** Data curation (lead); investigation (equal); methodology (equal); software (equal); validation (equal); writing – review and editing (supporting). **Jordan B. Sparks:** Project administration (lead); resources (lead); supervision (supporting); writing – review and editing (supporting). **Kristian D. Stensland:** Conceptualization (equal); methodology (equal); writing – review and editing (equal). **Zachery R. Reichert:** Conceptualization (equal); writing – review and editing (equal). **Joshi J. Alumkal:** Conceptualization (equal); writing – review and editing (equal). **Brent K. Hollenbeck:** Conceptualization (equal); methodology (supporting); writing – review and editing (equal). **Vahakn Shahinian:** Conceptualization (equal); methodology (equal); writing – review and editing (equal). **Alexander Tsodikov:** Methodology (supporting); visualization (supporting); writing – review and editing (supporting). **Ted A. Skolarus:** Conceptualization (supporting); funding acquisition (equal); methodology (equal); resources (supporting); supervision (supporting); writing – review and editing (equal).

## FUNDING INFORMATION

This work was funded by a Prostate Cancer Foundation Young Investigator Award. Drs. Skolarus and Caram are supported by National Cancer Institute grants R37CA222885 and R01CA242559. Dr. Tsodikov is supported by National Cancer Institute R01CA242559. Dr. Tsao is supported by the Prostate Cancer Foundation's Precision Oncology Program for Cancer of the Prostate. Dr. Stensland is supported by National Cancer Institute F32 CA264874. Drs. Hollenbeck, Shahinian, and Caram are supported by NCI R01 CA275993.

## CONFLICT OF INTEREST STATEMENT

J.J.A. has received consulting income from Fibrogen, Astellas, and Bristol Myers Squib and research support to his institution from Beactica, a Pfizer/Astellas/NCCN research award, and from Zenith Epigenetics.

## Supporting information


Data S1.

## Data Availability

A de‐identified, anonymized dataset will be created and shared. Where practicable, sharing should take place under a written agreement prohibiting the recipient from identifying or re‐identifying (or taking steps to identify or re‐identify) any individual whose data are included in the dataset. However, it is permissible for final datasets in machine‐readable format to be submitted to and accessed from PubMed Central (and similar sites) provided that care is taken to ensure that the individuals cannot be re‐identified using other publicly available information.
