# Supplementary material for: Survival by first‐line therapy and prognostic group among men with metastatic castration‐resistant prostate cancer
Source: Cancer Med. 2024 Jun 22;13(12):e7334. doi: 10.1002/cam4.7334 (PMC11193054; doi:10.1002/cam4.7334)
Supplement: Supplementary file 1 — Data S1. [file CAM4-13-e7334-s001.docx]

**Supplemental Figure. Final Cohort of Veterans with metastatic castration-resistant prostate cancer**

**Supplemental Figure Legend:** VA, Veterans Health Administration; ADT, Androgen Deprivation Therapy; PSA, Prostate Specific Antigen

^a^Treatment of inquiry: abiraterone, cabazitaxel, docetaxel, enzalutamide, ketoconazole, mitoxantrone, radium-223, sipuleucel-T

^b^Mitoxantrone: No patients received mitoxantrone as first-line treatment of inquiry

| **Supplemental Table 1.** Median and Interquartile Range (IQR) in the Total Cohort and Prognostic Groups | | | | |
| --- | --- | --- | --- | --- |
|  | **Total cohort (n=4,135)** | **Favorable (n=1,208)** | **Intermediate (n=1,527)** | **Poor (n=252)** |
| Hemoglobin (median and IQR) | 12.1 (10.6-13.3) | 13.3 (12.6-14.0) | 11.2 (10.1-11.9) | 9.5 (8.6, 10.4) |
| ALP (median and IQR) | 105 (75-189) | 84 (66-110) | 123 (79-234) | 419 (283-654) |
| Albumin (median and IQR) | 3.8 (3.4-4.1) | 4.0 (3.8-4.2) | 3.7 (3.3-4.0) | 2.9 (2.6-3.2) |

**Legend:** IQR, interquartile range; ALP, alkaline phosphatase

**Supplemental Table 2.** Patient Characteristics of Full Cohort, Prognostic Groups, and Patients with Missing Laboratory Data Who Were Not Included in Prognostic Groups

|  | **Full Cohort**  **(n = 4,135)** | **Patients with no missing labs (n=2,987)** | | |  | **Missing lab data (n=1,148)** | |
| --- | --- | --- | --- | --- | --- | --- | --- |
|  |  | **Favorable**  **(n = 1,208)** | **Intermediate**  **(n = 1,527)** | **Poor**  **(n = 252)** | **Group Com-parison p-value** | **ANY prognostic**  **labs missing**  **(n = 1,148)** | **ALL prognostic labs missing**  **(n = 400)** |
| **Drug (N, % by strata)** | | | | | <0.001 |  |  |
| Abiraterone | 1731 (42%) | 540 (45%) | 638 (42%) | 94 (37%) |  | 459 (40%) | 144 (36%) |
| Enzalutamide | 784 (19%) | 232 (19%) | 275 (18%) | 30 (12%) |  | 247 (22%) | 100 (25%) |
| Docetaxel | 762 (18%) | 218 (18%) | 344 (23%) | 89 (35%) |  | 111 (10%) | 26 (7%) |
| Ketoconazole | 858 (21%) | 218 (18%) | 270 (18%) | 39 (15%) |  | 331 (29%) | 130 (32%) |
| **Age at First Drug, years (median, IQR)** | 73  (66, 82) | 71  (66, 79) | 75  (67, 83) | 71  (65, 81) | <0.001 | 74  (67, 82) | 76  (67, 83) |
| **Race (N, % by strata)** | | | | | <0.001 |  |  |
| White | 2631 (64%) | 892 (74%) | 869 (57%) | 126 (50%) |  | 744 (65%) | 251 (63%) |
| Black | 1181 (29%) | 234 (19%) | 545 (36%) | 107 (42%) |  | 295 (26%) | 110 (28%) |
| Other | 68 (2%) | 17 (1%) | 30 (2%) | 3 (1%) |  | 18 (2%) | 7 (2%) |
| Unknown | 255 (6%) | 65 (5%) | 83 (5%) | 16 (6%) |  | 91 (8%) | 32 (8%) |
| **Charlson Comorbidity Index (N, % by strata)** | | | | | <0.001 |  |  |
| 0 | 2266 (55%) | 750 (62%) | 732 (48%) | 109 (43%) |  | 675 (59%) | 245 (61%) |
| 1 | 913 (22%) | 253 (21%) | 374 (24%) | 56 (22%) |  | 230 (20%) | 85 (21%) |
| 2+ | 956 (23%) | 205 (17%) | 421 (28%) | 87 (35%) |  | 243 (21%) | 70 (18%) |
| **Starting PSA, ng/mL (median, IQR)** | 46  (15, 144) | 25  (10, 68) | 63  (21, 188) | 344  (116, 810) | <0.001 | 41  (14, 117) | 35  (12, 102) |
| **PSA Doubling Time, months (median, IQR)** | 6.0  (4.7, 8.1) | 6.3  (5.1, 8.5) | 5.9  (4.6, 8.0) | 5.1  (4.0, 6.4) | <0.001 | 6.1  (4.8, 8.1) | 6.1  (4.7, 8.2) |
| **PSA Doubling Time (N, % by strata)** | | | | | <0.001 |  |  |
| < 3 months | 235 (6%) | 48 (4%) | 101 (7%) | 21 (8%) |  | 65 (6%) | 22 (6%) |
| 3 - <6 months | 1804 (44%) | 480 (40%) | 685 (45%) | 157 (62%) |  | 482 (42%) | 166 (42%) |
| 6 - <10 months | 1476 (36%) | 466 (39%) | 510 (33%) | 54 (21%) |  | 446 (39%) | 152 (38%) |
| ≥ 10 months | 620 (15%) | 214 (18%) | 231 (15%) | 20 (8%) |  | 155 (14%) | 60 (15%) |
| **Year at Time of First Treatment (N, % by year)** | | | | | <0.001 |  |  |
| 2010 | 346 | 108 (31%) | 118 (34%) | 27 (8%) |  | 93 (27%) | 33 (10%) |
| 2011 | 510 | 123 (24%) | 182 (36%) | 37 (7%) |  | 168 (33%) | 54 (11%) |
| 2012 | 457 | 99 (22%) | 190 (42%) | 42 (9%) |  | 126 (28%) | 41 (9%) |
| 2013 | 468 | 122 (26%) | 188 (40%) | 30 (6%) |  | 128 (27%) | 49 (10%) |
| 2014 | 548 | 172 (31%) | 202 (37%) | 33 (6%) |  | 141 (26%) | 36 (7%) |
| 2015 | 558 | 160 (29%) | 204 (37%) | 28 (5%) |  | 166 (30%) | 52 (9%) |
| 2016 | 605 | 209 (35%) | 208 (34%) | 31 (5%) |  | 157 (26%) | 73 (12%) |
| 2017 | 643 | 215 (33%) | 235 (37%) | 24 (4%) |  | 169 (26%) | 62 (10%) |
| **Distance to Facility, miles (median, IQR)** | 24  (9, 60) | 28  (11, 68) | 20  (8, 54) | 19  (7, 50) | <0.001 | 26  (9, 60) | 32  (10, 65) |
| Unknown (N, %) | 70 (2%) | 32 (3%) | 21 (1%) | 2 (1%) |  | 15 (1%) | 5 (1%) |
| **Complexity (N, % by strata)** | | | | | 0.033 |  |  |
| 1 (a + b + c) | 3523 (85%) | 1016 (84%) | 1321 (87%) | 224 (89%) |  | 962 (84%) | 302 (76%) |
| 2 | 433 (10%) | 135 (11%) | 151 (10%) | 24 (10%) |  | 123 (11%) | 66 (17%) |
| 3 | 161 (4%) | 52 (4%) | 47 (3%) | 2 (1%) |  | 60 (5%) | 29 (7%) |
| Unknown | 18 (0%) | 5 (0%) | 8 (1%) | 2 (1%) |  | 3 (0%) | 3 (1%) |
| **Rurality (N, % by strata)** | | | | | <0.001 |  |  |
| Urban | 2695 (65%) | 732 (61%) | 1042 (68%) | 188 (75%) |  | 733 (64%) | 239 (60%) |
| Rural | 1437 (35%) | 474 (39%) | 485 (32%) | 64 (25%) |  | 414 (36%) | 161 (40%) |
| Unknown | 3 (0%) | 2 (0%) | 0 (0%) | 0 (0%) |  | 1 (0%) | 0 (0%) |
| **HemOnc FTE (median, IQR)** | 2.63  (1.80, 3.68) | 2.60  (1.80, 3.68) | 2.63  (1.82, 3.87) | 2.60  (1.81, 4.43) | 0.9 | 2.75  (1.72, 3.66) | 2.24  (1.61, 3.66) |
| Unknown (N, %) | 21 (1%) | 6 (1%) | 9 (1%) | 1 (0%) |  | 5 (0%) | 4 (1%) |
| **HemOnc-Patient Ratio (median, IQR)** | 16  (14, 18) | 16  (14, 19) | 16  (14, 18) | 16  (13, 18) | 0.3 | 16  (14, 18) | 17  (13, 19) |
| Unknown (N, %) | 21 (1%) | 6 (1%) | 9 (1%) | 1 (0%) |  | 5 (0%) | 4 (1%) |
| **Urology FTE**  **(median, IQR)** | 2.15  (1.68, 3.52) | 2.16  (1.44, 3.28) | 2.15  (1.70, 3.28) | 2.03  (1.70, 2.89) | 0.2 | 2.17  (1.51, 3.71) | 1.98  (1.36, 3.28) |
| Unknown (N, %) | 21 (1%) | 6 (1%) | 9 (1%) | 1 (0%) |  | 5 (0%) | 4 (1%) |
| **Urology-Patient Ratio (median, IQR)** | 8.54  (6.66, 10.5) | 8.04  (6.66, 10.5) | 8.04  (6.57, 10.3) | 8.03  (6.57, 10.3) | 0.3 | 8.83  (7.21, 10.6) | 8.36  (6.63, 10.6) |
| Unknown (N, %) | 21 (1%) | 6 (1%) | 9 (1%) | 1 (0%) |  | 5 (0%) | 4 (1%) |
| **Early Treatment Adoption*, within 6 months (N, % by strata)** | | | | | 0.5 |  |  |
| Both Abi & Enza | 3306 (80%) | 933 (77%) | 1200 (79%) | 196 (78%) |  | 977 (85%) | 303 (76%) |
| Abi Only | 405 (10%) | 134 (11%) | 163 (11%) | 25 (10%) |  | 83 (7%) | 44 (11%) |
| Enza Only | 174 (4%) | 66 (6%) | 67 (4%) | 9 (4%) |  | 32 (3%) | 19 (5%) |
| Neither | 250 (6%) | 75 (6%) | 97 (6%) | 22 (9%) |  | 56 (5%) | 34 (9%) |
| **Proportion Treated (N, % by strata)** | | | | | 0.3 |  |  |
| Q1 (0.00% -0.04%) | 201 (5%) | 52 (4%) | 92 (6%) | 10 (4%) |  | 47 (4%) | 19 (4.8%) |
| Q2 (0.04% -0.08%) | 732 (18%) | 234 (19%) | 273 (18%) | 51 (20%) |  | 174 (15%) | 52 (13%) |
| Q3 (0.08% -0.13%) | 1219 (29%) | 379 (31%) | 489 (32%) | 73 (29%) |  | 278 (24%) | 115 (29%) |
| Q4 (0.13% -0.32%) | 1962 (47%) | 537 (44%) | 664 (43%) | 117 (46%) |  | 644 (56%) | 210 (53%) |
| Unknown | 21 (1%) | 6 (1%) | 9 (1%) | 1 (0%) |  | 5 (0%) | 4 (1%) |
| **Proportion Black (N, % by strata)** | | | | | <0.001 |  |  |
| Q1 (0.2% - 3.3%) | 575 (14%) | 168 (14%) | 168 (11%) | 22 (8.7%) |  | 217 (19%) | 76 (19%) |
| Q2 (3.3% - 9.0%) | 939 (23%) | 280 (23%) | 282 (18%) | 38 (15%) |  | 339 (30%) | 126 (32%) |
| Q3 (9.0% - 19.4%) | 1215 (29%) | 388 (32%) | 493 (32%) | 83 (33%) |  | 251 (22%) | 84 (21%) |
| Q4 (19.4% - 49%) | 1406 (34%) | 372 (31%) | 584 (38%) | 109 (43%) |  | 341 (30%) | 114 (28%) |

**Legend**: PSA, prostate specific antigen; IQR, interquartile range; FTE, full-time equivalent; Prognostic groups were defined as: Favorable group included patients who had hemoglobin >10 grams/deciliter (g/dl), albumin > 3.5 g/dl, and alkaline phosphatase < 200 International Unites/Liter (U/L) at the start of metastatic castration-resistant prostate cancer therapy; Intermediate group included patients with one or two of these laboratory values that was out of the favorable range; Poor prognostic group included patients for whom all three values were out of the favorable range. Facility variables are also included in this supplemental table. Facility complexity 1 is the most “complex” VA facility offering the most comprehensive care with complex clinical programs and research and teaching programs. Early treatment adoption was a novel facility level variable that assigned an adoption phenotype to facilities depending on whether a facility is an early versus a late adopter of newer therapies (i.e., abiraterone or enzalutamide). We expected facilities without access to specialty pharmacies or pharmacist expertise may systematically differ in quality, which could confound survival outcomes. Early treatment adoption variable was binary – to determine if a facility was an “early adopter, we assigned dates to each facility for when abiraterone and enzalutamide were first used at that site, following FDA approval. Facilities that adopted within six months of their first FDA approval were considered early adopters and all others later adopters. Proportion treated variable describes quartiles of facilities by the proportion of patients in that facility diagnosed with prostate cancer – this variable is a surrogate for patient volume with advanced prostate cancer. Proportion of Black variable describes the proportion of patients followed at that facility for any condition who are Black.

**Supplemental Table 3.** Multivariable Cox Regression Analysis for PSA Progression-Free Survival

| **Independent Variables** | **Hazard Ratios (95% Confidence Intervals)** | | |
| --- | --- | --- | --- |
|  | **Stratified Models (# of observations in full adjusted model)** | | |
|  | **Favorable**  **(n = 1,014)** | **Intermediate**  **(n = 1,185)** | **Poor**  **(n = 149)** |
| **First-line Treatment** | | | |
| Abiraterone | Ref. | Ref. | Ref. |
| Enzalutamide | **0.70 (0.55, 0.90)** | **0.78 (0.62, 0.97)** | 1.67 (0.69, 4.05) |
| Docetaxel | **1.67 (1.30, 2.15)** | 1.11 (0.90, 1.37) | 1.30 (0.69, 2.44) |
| Ketoconazole | **1.76 (1.34, 2.31)** | **1.78 (1.41, 2.25)** | **8.01 (2.93, 21.9)** |
| **Age (years)** | 1.00 (0.99, 1.01) | 0.99 (0.99, 1.00) | 0.99 (0.96, 1.01) |
| **Race** | | | |
| White | Ref. | Ref. | Ref. |
| Black | 0.94 (0.76, 1.16) | 0.96 (0.81, 1.14) | 1.11 (0.53, 2.32) |
| Other | 0.97 (0.45, 2.10) | 0.89 (0.57, 1.38) | 0.56 (0.05, 6.45) |
| **Charlson Comorbidity Index** | | | |
| 0 | Ref. | Ref. | Ref. |
| 1 | **1.33 (1.09, 1.61)** | 1.05 (0.89, 1.25) | 1.19 (0.57, 2.45) |
| 2+ | 1.07 (0.86, 1.33) | 0.91 (0.77, 1.08) | 1.06 (0.53, 2.10) |
| **Starting PSA (log scale)** | **1.19 (1.12, 1.26)** | **1.07 (1.03, 1.12)** | **1.41 (1.12, 1.77)** |
| **PSA Doubling Time** | | | |
| <3 months | Ref. | Ref. | Ref. |
| 3-6 months | 1.04 (0.68, 1.59) | 1.13 (0.84, 1.54) | 1.77 (0.76, 4.11) |
| 6-10 months | 0.77 (0.50, 1.17) | 0.89 (0.66, 1.22) | 1.72 (0.61, 4.87) |
| >10 months | 0.81 (0.52, 1.26) | **0.67 (0.47, 0.94)** | 1.05 (0.36, 3.05) |
| **Start Year** | | | |
| 2010 | Ref. | Ref. | Ref. |
| 2011 | 1.15 (0.83, 1.61) | 1.06 (0.77, 1.45) | 1.48 (0.58, 3.81) |
| 2012 | **1.57 (1.10, 2.24)** | 1.12 (0.82, 1.53) | 1.38 (0.52, 3.65) |
| 2013 | 0.97 (0.68, 1.38) | 1.10 (0.78, 1.54) | 2.39 (0.84, 6.79) |
| 2014 | 1.18 (0.82, 1.68) | 1.09 (0.77, 1.53) | **3.01 (1.08, 8.37)** |
| 2015 | 1.06 (0.72, 1.55) | 1.24 (0.88, 1.76) | 1.52 (0.55, 4.21) |
| 2016 | 1.25 (0.86, 1.81) | 1.00 (0.70, 1.43) | 2.03 (0.73, 5.63) |
| 2017 | 1.22 (0.80, 1.87) | 1.04 (0.70, 1.53) | 0.68 (0.16, 2.87) |
| **Distance to Facility (per 10 miles)** | 1.00 (0.99, 1.01) | 1.00 (0.99, 1.01) | 0.99 (0.98, 1.01) |
| **Urban or Rural** | | | |
| Urban | Ref. | Ref. | Ref. |
| Rural | 1.00 (0.84, 1.18) | 1.00 (0.85, 1.17) | 1.40 (0.71, 2.77) |

**Legend**: PSA, prostate specific antigen. The distance from the patient’s home zip code to the treating facility and urban/rural status of the facility are variables shown here. Urban/rural status was determined by the patient’s zip. Hazard ratios were also adjusted for facility level covariates including facility complexity, proportion of Black patients, proportion of patients with diagnosed prostate cancer who were treated for advanced disease, staffing of hematology/oncology and urology physicians (based on full-time equivalents), and whether the facility was an early adopter of novel oral therapies abiraterone and enzalutamide. These other variables not shown were not significant. *PSA was log-transformed for this multivariable analysis to make it more normally distributed. Prognostic groups were defined as: Favorable group included patients who had hemoglobin >10 grams/deciliter (g/dl), albumin > 3.5 g/dl, and alkaline phosphatase < 200 International Unites/Liter (U/L) at the start of metastatic castration-resistant prostate cancer therapy; Intermediate group included patients with one or two of these laboratory values that was out of the favorable range; Poor prognostic group included patients for whom all three values were out of the favorable range.

**Supplemental Table 4.** Subsequent Therapy After First-Line Metastatic Castration-Resistant Prostate Cancer Therapy (n=4,135)

| **Second-line Treatment** | **First-line Treatment** | | | |
| --- | --- | --- | --- | --- |
|  | Abiraterone (n=1,731) | Enzalutamide (n=784) | Ketoconazole (n=858) | Docetaxel (n=762) |
| None | 958 (55%) | 558 (71%) | 422 (49%) | 263 (35%) |
| Abiraterone | - | 178 (23%) | 246 (29%) | 373 (49%) |
| Enzalutamide | 587 (34%) | - | 40 (5%) | 73 (10%) |
| Ketoconazole | 7 (0%) | 3 (0%) | - | 29 (4%) |
| Docetaxel | 155 (9%) | 38 (5%) | 144 (17%) | - |
| Other | 24 (1%) | 7 (1%) | 6 (1%) | 24 (3%) |
